# Prospective Observational Study after Eversion Carotid Endarterectomy with Ultrasound-Guided Deep-Intermediate Cervical Plexus Blockade

**DOI:** 10.3390/healthcare10101986

**Published:** 2022-10-10

**Authors:** María Vega Colón, José Manuel López González, Bárbara María Jiménez Gómez, Jandro Pico Veloso, Marta Fernández Mendez, Félix Ezequiel Fernández Suárez, José Antonio del Castro Madrazo, Francisco Álvarez Marcos, Mario Fajardo Pérez, Jui-An Lin, Felice Galluccio, Jin-De Hou, Shun-Ming Chan

**Affiliations:** 1 Division of Cardiovascular and Thoracic Anesthesiology, Asturias University Central Hospital (HUCA), 33001 Oviedo, Spain; 2 Vascular Surgery Department, Asturias University Central Hospital (HUCA), 33001 Oviedo, Spain; 3 Morphological Madrid Research Center (MoMaRC), Ultradissection Spain Echo Training School, 28029 Madrid, Spain; 4 Center for Regional Anesthesia and Pain Medicine, Chung Shan Medical University Hospital, Taichung 40201, Taiwan; 5 Department of Anesthesiology, School of Medicine, Chung Shan Medical University, Taichung 40201, Taiwan; 6 Department of Anesthesiology, Chung Shan Medical University Hospital, Taichung 40201, Taiwan; 7 Department of Anesthesiology, School of Medicine, National Defense Medical Center, Taipei 11490, Taiwan; 8 Department of Anesthesiology, School of Medicine, College of Medicine, Taipei Medical University, Taipei 11031, Taiwan; 9 Center for Regional Anesthesia and Pain Medicine, Wan Fang Hospital, Taipei Medical University, Taipei 11696, Taiwan; 10 Division of Anesthesiology, Hualien Armed Forces General Hospital, Hualien 97144, Taiwan; 11 Department of Anesthesiology, Tri-Service General Hospital and National Defense Medical Center, Taipei 11490, Taiwan

**Keywords:** endarterectomy, carotid, endarterectomy: eversion, cervical plexus block: intermediate-deep, arterial pressure, oximetry: cerebral, sedation

## Abstract

(1) Introduction: The aim was to describe the anesthetic and surgical technique of eversion carotid endarterectomy performed under intermediate-deep cervical block with sedation, and to analyze the intraoperative and postoperative results. (2) Material and Methods: Thirty cases of unilateral eversion carotid endarterectomy (n = 30), performed between 2019–2020 in a tertiary center under intermediate-deep ultrasound-guided cervical plexus block and sedation, were prospectively observed and analyzed. Hemodynamic (blood pressure, heart rate) and neurological (cerebral oximetry) variables were measured in four intraoperative phases: at the beginning of the operation, prior to carotid clamping, after unclamping and at the end of the operation. We assessed acute postoperative pain in a numerical rating scale at 6, 12 and 24 h, early and 30-day complications, and length of stay. (3) Results: Baseline mean arterial pressure values were 100.4 ± 18 mmHg, pre-clamping 95.8 ± 14 mmHg, post-clamping 94.9 ± 11 mmHg, and at the end of the operation 102.4 ± 16 mmHg. Cerebral oximetry values were 61.7 ± 7/62.7 ± 8, 68.5 ± 9.6/69.1 ± 11.7 and 68.1 ± 10/68.1 ± 10 for the left and right hemispheres at baseline, pre- and post-clamping, respectively. The pain assessment showed a score less than or equal to 3. The incidence of residual nerve block, early complications, and major complications in the first 30 days was 40%, 16.7% and 3.3%, respectively. (4) Conclusions: The combination of intermediate-deep cervical plexus block and low-dose sedation is an effective and safe alternative in awake eversion carotid endarterectomy.

## 1. Introduction

Carotid endarterectomy is a standard surgical procedure that removes atheromatous plaque with stroke potential and repairs the artery afterward. This technique can be performed by placing an autologous or prosthetic patch to prevent restenosis or by severing the artery and removing the plaque for immediate reimplantation at the carotid bifurcation, following the eversion technique. Although the results of both surgical techniques are comparable in terms of short- and long-term outcomes, the eversion endarterectomy technique offers shorter clamping times and a lower risk of long-term infections due to the absence of prosthetic material [1].

Since the publication in 2008 of the GALA [2] clinical trial, many studies have been performed to determine which anesthetic technique offers the best short- and medium-term results [3,4,5,6]. However, no clinical trials have found significant differences between performing the procedure under general anesthesia and regional anesthesia (cervical plexus block). Although regional anesthesia techniques have been improved with the introduction of ultrasound guidance (superficial, intermediate and deep cervical plexus blocks [7]) and complementary sedation techniques, the best regional anesthesia plan with the best clinical results has not been defined. We may suppose that the anesthetic, hemodynamic and neurological results of carotid endarterectomy with a regional cervical block are similar to those performed under general anesthesia.

We present the results of a descriptive study on patients undergoing eversion endarterectomy under an intermediate-deep cervical block and mild sedation. The main objective is to assess the hemodynamic and neurological stability during surgery, to conduct a pain measurement, and to record complications in the immediate postoperative period.

## 2. Material and Methods

This was a single-center prospective observational study. No treatment or procedure performed differed from the center’s usual anesthetic and surgical protocol. Since 2019, all carotid endarterectomy procedures that reach pre-established criteria have been performed under intermediate-deep cervical plexus block and sedation, using the eversion endarterectomy technique. Exclusion criteria included a difficulty understanding or cooperating during surgery, an allergy to any drugs included in the protocol, a history or difficult airway predictors, and the presence of contraindications for regional anesthesia (coagulation disorders, infection, or hematoma next to the puncture site). All patients undergoing unilateral eversion carotid endarterectomy in 2019 and 2020 using intermediate-deep cervical plexus block and sedation were included in this study. The study was approved by the regional Medical Research Ethics Committee (CEImPA code 2021.159). All subjects were informed about the study, received written information about the study, and signed a consent form to participate.

The need to reduce scheduled surgical activity and the work overload derived from the COVID-19 situation led to an institutional change in anesthetic-surgical techniques that favored an intensified recovery protocol prioritization. In all cases, a PCR for COVID-19 detection was performed the day before surgery.

### 2.1. Anesthetic Technique

Patients were monitored with an electrocardiogram, invasive blood pressure, peripheral oxygen saturation, and neurological monitoring of the anesthetic depth using a bispectral index monitor (bilateral BIS, A-2000TM Version 3.4; Aspect Medical System Inc., Norwood, MA, USA) and cerebral oximetry using an INVOS 3100 monitor (Somanetics Corp^®^ Medtronic, Minneapolis, MN, USA). All patients were premedicated with 0.03 mg/kg midazolam, and sedoanalgesia (remifentanil 0.03–0.05 mcg/kg/min or dexmedetomidine 0.5–0.7 mcg/kg/h) was applied, adjusted for patient comfort and allowing patient cooperation. All patients underwent cervical plexus block after premedication using a portable M-Turbo ultrasound machine (Sonosite^®^, Bothell, WA, USA) with a high-frequency linear transducer 6–15 MHz and Echoplex^®^ 22G 50 mm needle (Vygon, Ecouen, France).

**Figure 1:**
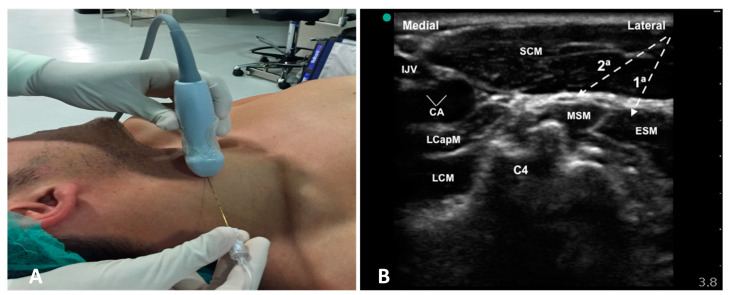
Ultrasound-guided cervical plexus block. (**A**) Position of the ultrasound probe and puncture site at the carotid bifurcation. (**B**) Ultrasound image of the deep (1st) and intermediate (2nd) cervical plexus block. SCM: sternocleidomastoid muscle; LCM: longus colli muscle; LCapM: longus capitis muscle; MSM: middle scalene muscle; ESM: elevator scapulae muscle; IJV: internal jugular vein; CA: carotid artery bifurcation; C4: C4 vertebra transverse process.

The probe was positioned at C4 (the bifurcation of the carotid artery is at this level in most patients) (Figure 1A). The superficial cervical plexus appears as a small hypoechoic (honeycomb-like) structure immediately deep or lateral to the posterior border of the sternocleidomastoid muscle (SCM) (Figure 1B). However, they are not always so evident in the intermediate plane, between the superficial and the deep cervical fascia.

The puncture is performed in-plane, laterally to medially, visualizing the needle until it passes the deep cervical fascia (prevertebral sheet) that covers the deep neck musculature. A total of 20 mL of levobupivacaine 0.25% was administered in two injection sites (Figure 1B); the first (6 mL) was administered to block the nerve branches of the deep cervical plexus, before withdrawing to the intermediate plane, where the second injection (14 mL) was made to block the nerve branches of the superficial cervical plexus. During the procedure, if the patient reported pain or discomfort after the skin incision or when entering the adventitia surrounding the vessels, it was reinforced with local infiltration of lidocaine 2% by the surgical team through the wound.

### 2.2. Surgical Technique

**Figure 2:**
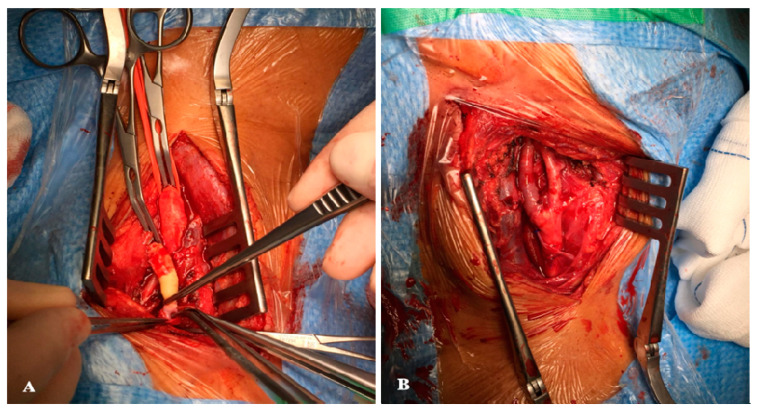
Carotid endarterectomy by eversion. (**A**) Transverse arteriotomy during endarterectomy by eversion with removal of the atheroma plaque. (**B**) Final reconstruction of the carotid bifurcation after eversion.

Using an oblique longitudinal incision along the medial edge of the sternocleidomastoid muscle, dissection was performed in planes using an electric scalpel until the plane of the carotid axis was located. At this point, a dose of intravenous anticoagulant sodium heparin was administered at a rate of 1 mg/kg. The carotid sector was exposed from 3–4 cm proximal to the bulb until the lesion in the internal carotid artery was comfortably overlapped. At this point, a test clamp of the internal carotid artery was performed for 1 min. If, during this minute, the patient does not present neurological deterioration, the planned procedure is continued (if neurological symptoms are present, the endarterectomy technique with a patch and shunt placement is chosen). After clamping, a complete oblique section of the internal carotid artery was performed, covering part of the carotid bulb and the resection of the atheromatous plaque by eversion (Figure 2). Subsequently, after the revision and washing of the endarterectomy areas, the internal carotid artery was reimplanted at its origin using a continuous 6/0 nylon suture. Finally, angiography control was performed to check for the absence of defects in the endarterectomized area of the internal carotid artery by a pigeonhole puncture in the common carotid artery. Protamine sulfate was administered to reverse the state of anticoagulation, hemostasis was checked, and the surgical wound was closed in planes with the placement of an aspiration drain [8].

The primary objectives included the monitoring of hemodynamic variables (blood pressure and heart rate) and neurological variables (cerebral oximetry) in four phases of the intraoperative period: baseline, prior to carotid clamping (2 min before), after unclamping (2 min after), and at the end of the operation. In the secondary objectives, the pain was assessed using a numerical rating scale (NRS) from 0 to 10 at 6, 12, and 24 h, and short-term complications were recorded in the first 24 h post-intervention (hematoma, bleeding, cough, transient nerve blocks, reperfusion syndrome, and stroke) and cardiovascular complications over 30 days (acute myocardial infarction, acute stroke, and mortality), as well as the length of stay (both in the resuscitation unit and the hospital stay). The transient residual block was assessed during the immediate postoperative period by neurological examination of the patient, assessing whether the following cranial nerves were affected: VII (facial paralysis), X (dysphonia), and XII (tongue deviation).

### 2.3. Statistical Analysis

Continuous variables were described by mean and standard deviation, using the median and range according to the distribution. Discrete variables were expressed as the absolute frequency and relative percentage. The normality distribution was tested using the Shapiro-Wilk test. Given the limited sample size and the absence of a control group, the analysis remained exclusively descriptive. All calculations were performed using SPSS version 15.0 software (IBM Corporation, Armonk, NY, USA).

## 3. Results

Thirty consecutive patients were included in the study. Demographic and background data are shown in Table 1. Interventions were 50% right-sided and 50% left-sided, and sedation was performed with remifentanil (51%) or with dexmedetomidine (49%). Most patients included in the study were male (73.3%), older than 70 years, and with an anesthetic risk scale score of ASA III (Table 1). In more than 70% of patients, the intermediate-deep cervical block was effective without requiring intraoperative reinforcement. The mean operative times were 98.4 ± 16 min, and the carotid clamping times were 19.9 ± 5 min.

**Figure 3:**
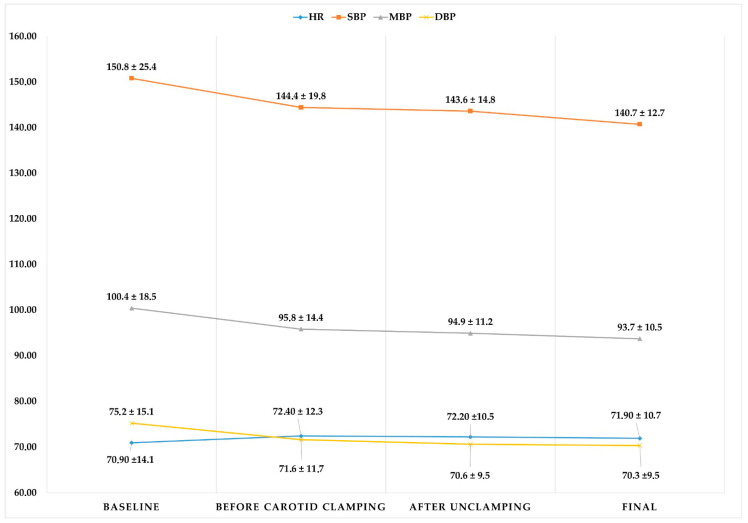
Hemodynamic variability during the intervention expressed as heart rate and blood pressure values. HR, heart rate; SBP, systolic blood pressure; MBP, median blood pressure; DBP, diastolic blood pressure. Data are expressed as mean and standard deviation.

**Figure 4:**
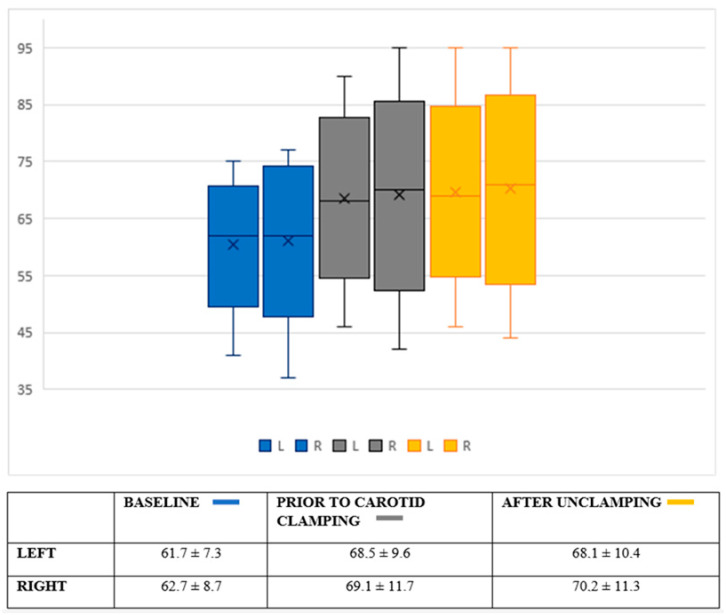
Box plot representation of the variability of cerebral oximetry (INVOS 3100 system, Somanetics Corp^®^ Medtronic, Minneapolis, MN, USA). Values expressed in the table as mean and standard deviation.

There was little intraoperative hemodynamic variability (Figure 3), with mean arterial blood pressure (MAP) values remaining around 90–100 mmHg during the operation (initial values of 100.4 ± 18 mmHg, 95.8 ± 14 mmHg prior to arterial clamping, and 94.9 ± 11 mmHg after unclamping). This stability was also seen in the heart rate, whose mean values remained between 70.9–72.4 beats per minute throughout the procedure. The cerebral oximetry values (Figure 4) and BIS values (Table 2) also remained stable. The mean cerebral oximetry value was 61.7 for the left and 62.7 for the right hemispheres. After locoregional blockade and before carotid clamping, there was a slight increase in the mean oximetry value. The value with the most significant variability was reached before arterial clamping at the right hemisphere but was without clinical significance.

Among the results collected in the postoperative period were the following: reasonable pain control with NRS values below 3/10 during the first 24 h; an overall incidence of a transient residual nerve block (40%) mainly concerning the laryngeal and hypoglossal nerves; and 16.7% with early complications, the main one being cervical hematoma (6.7%). Management of these complications was conservative, except in one case requiring reoperation. The incidence of major complications in the first 30 days was one stroke. The median length of stay in the resuscitation unit was 24 h, and the median length of hospital stay was 60 h (Table 3).

## 4. Discussion

The present study shows promising results for CEA by eversion under cervical plexus block and light sedation. Our study shows excellent surgical and carotid clamping times and maintains hemodynamic and cerebral oximetry stability during the procedure. Moreover, it also shows acceptable postoperative complication rates (transient residual block 40%, cervical hematoma 6.7%).

Carotid endarterectomy is the treatment of choice to reduce the risk of stroke in patients with significant carotid stenosis [9,10]. However, in the last decade, carotid stenting has gained popularity as an alternative technique in patients with high surgical risk associated with contraindication to endarterectomy, stenosis in sites inaccessible for surgery, restenosis after the previous endarterectomy, previous surgery, radiation to the neck, or concomitant severe cardiac pathology [11]. There is no current consensus on which technique to choose. However, both alternatives should be considered to be complementary, and the choice should be based on clinical criteria and the experience of the care teams [12].

There is also no consensus on the anesthetic technique of choice. Following the publication of the GALA2 study, which found no differences between general and regional anesthesia in terms of mortality, perioperative stroke, and acute myocardial infarction, many studies have been published that try to determine which anesthetic technique offers the best short- and medium-term results. Despite not finding significant differences between both anesthetic techniques, real-time neurological monitoring with an awake patient is an advantage that favors the use of regional anesthesia techniques. Moreover, some studies describe a better hemodynamic stability and preservation of cerebral blood flow during surgery, fewer hemorrhagic and cardiorespiratory complications, and a better control of acute postoperative pain [13,14].

Hemodynamic variability during carotid endarterectomy has been extensively studied in recent years. Hoefer et al. [15] observed an increase in the mean systolic blood pressure and the release of stress hormones (cortisol, metanephrine and normetanephrine) after performing an intermediate cervical block. Despite using a similar sample size (32 patients vs. 30 patients) and having a similar hypertension incidence (68.8% vs. 76.7%), Hoefer et al.’s results contrast with ours. There are two fundamental differences between both studies. The first one is the hemodynamic variable collected, since they only obtained differences in the systolic blood pressure and did not register the mean arterial pressure. The second difference is the study design: they performed an intermediate cervical plexus block, possibly responsible for less sympathetic block compared to the one achieved by the intermediate-deep block we performed. However, more studies will be needed to support this hypothesis.

Regarding the postoperative pain and transient nerve block, Kavakli et al.’s [7] study is worth mentioning. These two variables were studied after carotid endarterectomy in relation to a combined (superficial and deep) or intermediate cervical blockade. The blockades were performed with bupivacaine 0.5% with a mean volume of 28.7 ± 4.4 mL for the combined cervical block and 25.6 ± 6.2 mL for the intermediate cervical block, both being higher than ours (all patients received 6 mL for the deep cervical plexus block and 14 mL for the intermediate cervical plexus block with levobupivacaine 0.25%). The surgery times were similar (86 ± 12 vs. 98.4 ± 16 min). However, higher peak NRS values were recorded in the first 24 h postoperatively, reaching statistical significance (2.2 for the combined cervical block; 5.3 for the intermediate block; *p* = 0.044). In the present study, NRS values remained below a score of 3/10 during the first 24 h after surgery. These differences could be explained by the different ultrasound-guided cervical plexus block techniques and injection sites used.

An improvement in the cerebral oximetry values from the beginning of the intervention was registered, probably due to local vasodilatation secondary to the blockade. This phenomenon was also used by Malik et al. [16] to explain the decrease in the transfusion rate observed in their study. However, one cannot rule out the hypothesis that the decrease in cerebral oxygen consumption is related to the intraoperative use of sedative drugs.

On the other hand, this vasodilatation, as hypothesized by Malik et al. [16] in their work, could justify the lower incidence of bleeding in the group undergoing regional anesthesia.

One of the fundamental postoperative variables is the incidence of postoperative transient nerve block in terms of morbidity and patient discomfort [17]. After this surgery, there was a higher incidence of hoarseness due to transient laryngeal nerve block. It varied widely, from 12% in the intermediate cervical block group and 17% in the combined cervical block group in the Kavakli et al. [7] study to 75% in the Hoefer et al. study [15] with intermediate cervical block. Although the incidence of laryngeal nerve block in this work was intermediate (23%), the most frequent postoperative residual nerve block that was found was that of the hypoglossal nerve (30% of patients). On the other hand, in our study the incidence of major complications (stroke) at 30 days was low (3.3%), very similar to that of the GALA2 study (3.7%) and of Hoefer et al. [15] (3.1%). In the Leichtle et al. [5] and Crest [18] studies, the incidence was lower (2.38% and 1.8%, respectively).

In some cases, it may be necessary to convert from locoregional to general anesthesia during the procedure [19]. The main causes are the failure of the regional technique, claustrophobia, airway obstruction secondary to cervical hematoma, diaphragmatic or vocal cord paralysis causing respiratory distress, loss of consciousness during carotid clamping, and complications related to shunt placement (inadequate cerebral perfusion, embolization of air or atheroma plaques, mechanical problems during insertion or removal) [20,21]. Despite all these possible causes, the published conversion rates are very low (4 out of 2463 patients in the study by Pasin et al. [22]), consistent with the results described herein, where no patient required conversion.

The present study has several limitations that should be noted. One of the main ones is the absence of satisfaction surveys on patient comfort during the procedure and the fact that in most of the previous studies with regional techniques, anatomical references are used for their performance or the surgeon performs the infiltration under direct vision, which makes a comparison difficult. On the other hand, the lack of publications comparing intraoperative cerebral oximetry values in carotid endarterectomy under general anesthesia and regional anesthesia is a limitation to drawing conclusions from these findings. We also highlight the two different sedation regimens used (remifentanil and dexmedetomidine), which could represent a bias due to their different mechanisms of action, hemodynamic profiles, influences on postoperative pain, and neurocognitive outcomes. However, we would like to point out that shallow doses of both drugs are used for the comfort of the awake patient during the operation, as a result of which the hemodynamic and neurological influence is minimal and no complications arising from their use have been reported.

## 5. Conclusions

Our study suggests that performing an ultrasound-guided deep intermediate cervical plexus block with a continuous low-dose sedation (remifentanil or dexmedetomidine) appears to be a safe and effective alternative to the anesthetic management of carotid endarterectomy by eversion in an awake patient. Further larger cohort studies will be needed to confirm these results.

## Figures and Tables

**Table 1:** Demographic data and comorbidities. Data are expressed as percentages and as mean +/− SD. ASA, American Society of Anesthesiology; COPD, chronic obstructive pulmonary disease.

Variables	Sample Size (N = 30)
Men	22 (73.3%)
Women	8 (26.7%)
Age (years)	71.2 ± 8.7
Weight (kg)	76.5 ± 12.7
Height (cm)	166.0 ± 9.56
ASA ASA III or superior	2.9 ± 0.2 28 (93.3%)
Comorbidities	
Hypertension	23 (76.7%)
Diabetes mellitus	11 (36.7%)
Atrial fibrillation	8 (26.7%)
Ischemic heart disease	4 (13.3%)
Dyslipidemia	19 (63.3%)
Transient ischemic attack	21 (70%)
Stroke	16 (53.3%)
Active smoker	18 (60%)
Hypothyroidism	2 (6.7%)
Sleep apnea syndrome	1 (3.3%)
Chronic renal syndrome	3 (10%)
COPD	3 (10%)
Contralateral stenosis	11 (36.7%)

**Table 2:** Values of bispectral index monitor during surgery (bilateral BIS, A-2000TM Version 3.4; Aspect Medical System Inc., Norwood, MA, USA). Values expressed in the table as mean and standard deviation for normal variables and median and range for non-normal ones.

Baseline	After Anesthesia	Final
97.5 (97–98)	85.6 ± 3.8	96.4 ± 1.3

**Table 3:** Postoperative pain characteristics expressed through the numerical rating scale (NRS), incidence of transient residual nerve block, early and major complications, and length of stay. Data are expressed as percentages and as mean +/− SD. Length of stay values expressed as median and range.

Variable	Sample Size (N = 30)
Postoperative pain	
6 h	0.4 ± 0.9
12 h	1.2 ± 0.9
24 h	0.47 ± 0.5
Transient residual nerve block	12 (40%)
Hypoglossal nerve block	9 (30%)
Laryngeal nerve block	7 (23.3%)
Facial nerve block	2 (6.7%)
Early complications	5 (16.7%)
Cervical hematoma	2 (6.7%)
Bleeding	1 (3.3%)
Pharyngeal edema	1 (3.3%)
Headache	1 (3.3%)
Major complications in the first 30 days	1 (3.3%)
Length of stay at resuscitation unit (h)	24 (24–24)
Hospital length of stay (h)	60 (48–78)

## Data Availability

The datasets generated and analyzed during the current study are available from the first author on reasonable request.
